# Psychosocial and behavioral factors associated with HIV among adolescent girls and young women in DREAMS districts in South Africa: cross-sectional survey

**DOI:** 10.3389/fpubh.2025.1620084

**Published:** 2025-08-26

**Authors:** Kaymarlin Govender, Sean Beckett, Olivier Mukuku, Cherie Cawood, Gavin George, David Khanyile, Tarylee Reddy, Richard Cowden, Adrian Puren

**Affiliations:** ^1^Health Economics and HIV and AIDS Research Division (HEARD), University of KwaZulu-Natal, Durban, South Africa; ^2^Institut Supérieur des Techniques Médicales de Lubumbashi, Lubumbashi, Lubumbashi, Democratic Republic of Congo; ^3^Epicentre AIDS Risk Management (Pty) Limited, Cape Town, South Africa; ^4^Biostatistics Research Unit, South African Medical Research Council (SAMRC), Durban, South Africa; ^5^Human Flourishing Program, Harvard University, Cambridge, MA, United States; ^6^South African National Institute for Communicable Diseases, Johannesburg, South Africa

**Keywords:** adolescent girls and young women, HIV, psychosocial risk, mental health, dreams, South Africa

## Abstract

**Background:**

High HIV prevalence among adolescent girls and young women (AGYW) in South Africa is driven by multiple interacting psychosocial risks. Some of these risks include socioeconomic deprivation, poor educational outcomes, poor mental health, alcohol and drug use which are associated with HIV-infection and sexual risk behavior. This article seeks to deepen our understanding of the role of psychosocial and behavioral factors in the HIV epidemic among AGYW.

**Methods:**

A cross-sectional survey (*n* = 18,296) targeting AGYW aged between 12 and 24 years old was undertaken in four districts in KwaZulu-Natal and Gauteng. The analysis used descriptive statistics and hierarchical multiple binary logistic regressions that were disaggregated by age.

**Results:**

The mean age is 18 (SD:4) years old. Displaying depressive symptoms had increased odds of being HIV positive among the 15-19-year-old age group of AGYW (AOR: 1.12, 95% CI: 1.05–1.18, *p* < 0.001). For the 20-24-year olds, increased substance use was associated with a higher likelihood of being HIV positive (AOR: 1.10, 95% CI: 1.03–1.17, *p* < 0.01) and those who experienced Intimate Partner Violence (IPV) were more likely to be HIV positive (AOR: 1.41, 95% CI: 1.08–1.85, *p* < 0.05). Our findings indicate a lower prevalence of depressive symptoms in the two adolescent groups (12–15 years old and 15–19 years old) than in the older group (20–24 years old); nevertheless, depressive symptoms increased the likelihood of being HIV positive.

**Conclusion:**

This study suggests that HIV interventions are likely to be beneficial if they address individual HIV risk factors such as poor mental health challenges and IPV. However, due to the cross-sectional design, causality or temporal sequencing—particularly in bidirectional relationships such as depression and HIV—cannot be established. Interpretations should therefore be made with caution.

## 1 Introduction

Among world regions, Eastern and Southern Africa (ESA) continues to have the highest burden of HIV/AIDS (1). In 2020, approximately 38 million people were living with HIV (PLHIV) globally, and Eastern and Southern Africa (ESA) accounted for 45% of all new HIV infections, despite representing only about 6% of the global population (2). Within ESA, the epidemic in South Africa remains one of the largest worldwide, with an estimated 7.8 million PLHIV in 2021, accounting for nearly 20% of the global HIV burden (2). Evidence over the past decade suggests that HIV prevalence in South Africa has stabilized and incidence has gradually declined (3), although declines remain insufficient and national estimates mask important age-sex heterogeneity. In South Africa, the prevalence of HIV among AGYW is up to two times higher than that of their male peers (3). A significant proportion of new HIV infections occur among adolescent girls and young women (AGYW) aged 15–24. In 2020, despite representing only about 10% of the population in Eastern and Southern Africa, AGYW accounted for 26.5% of all new HIV infections (2).

Several factors drive the high HIV prevalence and incidence among AGYW. Women are biologically more susceptible to acquiring HIV than men. Biological risk factors that increase susceptibility of AGYW to HIV transmission include cervical ectopy (which is particularly pronounced at younger ages), high levels of genital inflammation, and the presence of co-infections such as other sexually transmitted diseases (4, 5). HIV disparities are also driven by multiple interacting psychosocial risks experienced by AGYW, such as high rates of socioeconomic deprivation, which are associated with HIV-infection and sexual risk behavior (6, 7). Psychosocial factors can also be the outcome of an HIV diagnosis, for example someone may experience depression after contracting HIV.

In South Africa, poverty is a major concern in most families created by historical and current unequal cultural, social and economic status, and due to family’s economic deprivation AGYW are often expected to fend for themselves and to contribute financially or materially to support their families (7–9). The low socio-economic status of women reinforces unequal gender power dynamics. Consequently, among young women, low wealth is associated with unsafe sexual behaviors, including earlier sexual debut, multiple sexual partners (8), and lower likelihood of condom use at last sex (7, 8), as well as experiences of physically forced sex (9). Societal norms that support male superiority increase vulnerability of AGYW to HIV through creating unequal power dynamics that increase intimate partner violence perpetration and cause females to be unable to negotiate safe sex (10, 11).

Sociocultural and economic factors impede access to basic education for girls, as traditional perceptions on the expected roles of girls to be at home, do household chores and get married early persist (12). Consequently, girls fare much worse than boys in terms of school retention, completion, and performance rates (12, 13). Several studies report that education has a protective effect on HIV infection (14, 15) and currently, keeping girls in school is considered one of the most effective prevention methods for reducing HIV among adolescent girls across Africa (16). Adolescent girls also face a higher risk of depression than boys, partly due to greater exposure to interpersonal stressors such as sexual abuse, violence, and gender inequities (17). Globally, AGYW bear a disproportionate burden of depression (17), which is also prevalent in South Africa (18, 19). Depressive symptoms are linked to behaviors and relationship dynamics that increase HIV risk (20). Larsen et al. (21) found that over 10% of AGYW had moderate-to-severe depression (MSD), which was associated with frequent HIV risk behaviors. Women with depressive symptoms are more likely to report intimate partner violence (IPV), relationships with older partners, and transactional sex (22). Among HIV-positive individuals, severe depression is associated with delayed testing and entry into care (23).

Alcohol and other drugs are reported to play a major role in sexual risk taking as they interfere with judgment and decision making, and lead to feelings of reduced sexual control (24, 25). Marlow et al. (26) state that youth are more likely to experiment with alcohol and other drugs prior to sexual encounters, impairing their judgment and impulse control, which facilitates risky sexual behavior. Studies have shown than risky drinking is associated with intentions to engage in unprotected sex (27). On the contrary, low risk alcohol drinking was found to have a protective effect against HIV infection among young women (28).

Given the disproportionate burden of HIV among adolescent girls and young women (AGYW), particularly in socioeconomically disadvantaged contexts, targeted prevention initiatives are essential. One notable example is the DREAMS (Determined, Resilient, Empowered, AIDS-free, Mentored, and Safe) initiative—a comprehensive global program launched in 2015 by the U. S. President’s Emergency Plan for AIDS Relief (PEPFAR). The initiative aims to reduce HIV incidence among AGYW aged 15–24 years in 10 sub-Saharan African countries. DREAMS employs a multifaceted approach, combining evidence-based interventions such as condom promotion, HIV testing and counseling, and community mobilization to shift harmful social norms. By addressing structural and psychosocial drivers of risk, DREAMS provides a holistic and context-specific strategy to improve health outcomes among AGYW (29).

While previous studies have explored psychosocial and behavioral HIV risk factors among AGYW in South Africa (7–9), many used small or localized samples. This study fills a critical gap by leveraging a large, geographically diverse dataset from DREAMS districts, allowing for more nuanced understanding of contextual variations and broader generalizability. It provides a context-specific analysis incorporating localized socio-economic and community factors influencing HIV susceptibility.

Although psychosocial factors like depression and IPV are often considered predictors of HIV acquisition, the relationship is likely bidirectional; an HIV diagnosis itself may exacerbate depression and IPV experiences, complicating causal interpretations. This complexity is critical for research and intervention design. Our cross-sectional design limits causal inference, but the study aims to investigate psychosocial and behavioral correlates of HIV risk among AGYW in DREAMS districts. Special attention is given to high-risk subgroups such as young mothers and AGYW engaged in sex work. By delineating distinct risk profiles, the findings can inform tailored and effective HIV prevention strategies to address this population’s disproportionate burden.

## 2 Methods

### 2.1 Study setting

The study was conducted in four districts across two provinces in South Africa: Johannesburg and Ekurhuleni in Gauteng Province, and eThekwini and uMgungundlovu in KwaZulu-Natal Province. Survey data were collected from March 13, 2017 to June 22, 2018. More detail on the study setting can be found the study’s protocol paper (29).

### 2.2 Study design and sampling strategy

This population-based cross-sectional survey focused on adolescent girls and young women (AGYW) aged 15–24 years, and applied a stratified, multistage cluster sampling approach. The primary strata consisted of four districts: the City of Johannesburg and Ekurhuleni (Gauteng province), and eThekwini and uMgungundlovu (KwaZulu-Natal province) (29).

To achieve the target sample size of 18,500 AGYW—comprising 10,500 in Gauteng and 8,000 in KwaZulu-Natal—a total of 1,050 Small Area Layers (SALs) were randomly selected from a national sampling frame. SALs were distributed across districts based on population estimates and required statistical precision: 300 SALs each in Johannesburg, Ekurhuleni, and eThekwini, and 150 in uMgungundlovu. Despite its smaller population, uMgungundlovu was oversampled to ensure robust district-level estimates (29).

Within each selected SAL, 55 households were randomly sampled using GPS-assisted enumeration. This number was derived by accounting for an estimated 50% household eligibility rate, 20% anticipated non-response, and approximately 12.5% of households being vacant or unreachable. All AGYW aged 15–24 years who met the inclusion criteria and provided consent or assent were invited to participate (29).

In total, 18,296 AGYW were successfully enrolled, yielding a response rate of 98.9%. Sampling weights were applied to adjust for unequal selection probabilities, household non-response, and to enable population-level estimates at the provincial and district scales. The SAL sampling frame was constructed by triangulating data from the 2011 national census, the 2007 Community Survey, and recent satellite imagery, allowing for accurate population and household estimates disaggregated by district, age, sex, and race (29).

Sample size determination was guided primarily by the objective of detecting changes in HIV incidence. In KwaZulu-Natal, a sample of 8,000 AGYW provided 80% power to detect a 40% reduction in incidence from a baseline of 4 per 100 person-years (py), assuming 25% HIV prevalence, a 0.01% false-recent rate, and a 5% alpha level. In Gauteng, the sample of 10,500 was designed to detect a similar reduction from an estimated baseline incidence of 3 per 100 py under the same assumptions (29).

These sample sizes also support incidence estimation with acceptable precision. The KwaZulu-Natal sample enables estimation of a 4% incidence rate with a 12% coefficient of variation (CV), yielding a 95% confidence interval (CI) of 3.1–4.9%. For Gauteng, a 3% incidence rate can be estimated with the same CV and a 95% CI of 2.28–3.72% (29).

**Figure 1:**
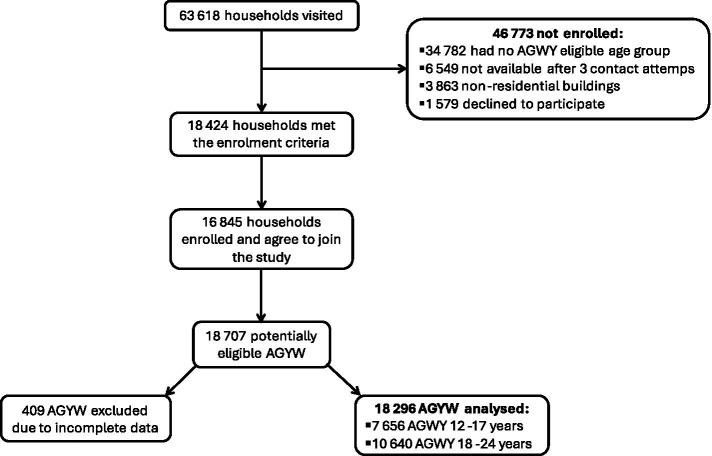
Participant flowchart for the study on HIV risk among AGYW in DREAMS districts, South Africa.

Household composition forms were completed by 63,618 caregivers. Among these, 18,424 households were eligible, and 16,845 consented to participate (Figure 1). Data collection involved two structured instruments administered by trained fieldworkers using personal digital assistants (PDAs): a caregiver questionnaire and an AGYW questionnaire, both adapted from the HIV Incidence Provincial Surveillance System (HIPSS). These tools captured information on demographics, household socioeconomic status, psychosocial well-being, behavioral and health-related characteristics, and exposure to DREAMS interventions. The questionnaires were developed in collaboration with community stakeholders and underwent pretesting prior to field deployment (29).

### 2.3 Ethical considerations

Written consent was obtained from all adolescent girls aged 18 years old and parental or guardian consent and individual written assent was obtained from all adolescent girls who were younger than 18 years. The study was granted ethical clearance by the University of KwaZulu-Natal Biomedical Research Ethics Committee (BFC 198/16) and the Provincial Department of Health in both KZN and GP. The study was performed in accordance with the ethical standards as laid down in the 1964 Declaration of Helsinki and its later amendments.

### 2.4 Measures

HIV diagnosis was determined through laboratory testing of blood samples obtained via finger prick and collected into BD Microtainer® blood collection tubes (Becton Dickinson, South Africa), with HIV antibody testing performed according to standard protocols.

Resilience was assessed using the shortened 12-item version of the Child and Youth Resilience Measure (CYRM-12) (30), which captures personal and social resilience resources such as peer support, problem-solving, and access to education and healthcare services.

Depressive symptoms were evaluated using the 5-item short form of the Center for Epidemiological Studies Depression Scale (CES-D-5) (31), which includes items reflecting feelings of sadness, hopelessness, loneliness, depressed mood, and sleep disturbance experienced in the past week.

Alcohol use was measured using the Alcohol Use Disorders Identification Test (AUDIT-C) (32). Substance use was measured by using a self-constructed checklist of 5 types of drugs. HIV knowledge was measured using 6 items testing individuals’ knowledge on how HIV is prevented (for, e.g., can people reduce their chances of contracting HIV by using a condom every time they have sex). Intimate Partner Violence (IPV) assessed the incidence of sexual and physical IPV, if the AGYW have experienced one of the 13 IPV instances, they were coded as having experienced IPV (33).

A measure on whether a family member has left the household in the previous 12 months for more than one continuous month was included in the study. A measure on food security was included in the study that asked whether in the past 4 weeks if there was no food to eat in the house and the response options were always, often, sometimes, never. Total household income in South African Rands was asked of the caregiver in the study. A measure on current school attendance was included in the study. The study also included a measure on whether AGYW have a safe place for them and their friends to meet in the community. The study also included a measure on whether AGYW attended a sports club. The study also asked how many of the respondent’s friends had consumed alcohol and how many had consumed marijuana.

### 2.5 Data analysis

Data analysis was undertaken using SPSS version 27. Our analysis used descriptive statistics and hierarchical multiple binary logistic regressions. Systemic level predictors of HIV status were entered into a hierarchical regression. Variables were entered into the model in successive blocks. We started with the most proximal level variables (individual level) and ended with the most distal level variables (community level). In addition, we disaggregated this by age of the AGYW. We present Adjusted Odds Ratios (AOR) with 95% confidence intervals (CI) for the multiple logistic regressions. To adjust for the study design and non-response, sampling weights were used. The final sampling weight was the product of the SAL weight, household weight, adjusted for individual non-response. The final individual weights were benchmarked against the 2018 Statistics South Africa mid-year population estimates by age group and province (34). Taylor series linearization methods were used to estimate standard errors. In all analyses, standard errors were adjusted for clustering by SAL.

## 3 Results

The mean age is 18 [standard deviation (SD):4] years old and the vast majority of the AGYW considered themselves African in the study sample. Only one-tenth 10.1% of the respondents had only primary school education. Only 6.3% have been away from home for more than one consecutive month in a year. Nearly two-thirds (65.3%) of households between 1,001 rands to 5,000 rands per month. Only just over a quarter (26.0%) of AGYW indicated that they never had a shortage of food to eat in their house (Table 1).

**Table 1:** Socio-demographic and psychosocial characteristics for DREAMS sample, 2017–2018.

Variable		Number	Mean (SD)/%
Age			18 (4)
Race	African	17,931	97.6%
	Colored	329	2.2%
	White	4	0.0%
	Asian/Indian	22	0.1%
	Other	10	0.1%
Highest education achieved	Primary	1955	10.1%
	High	14,201	89.9%
Length lived in their community	Always	16,110	87.4%
	Moved here less than 1 year ago	531	3.0%
	Moved here more than 1 year ago	1,650	9.6%
	Refused	5	0.0%
In the previous 12 months, been away from home for more than one consecutive month	No	17,217	93.6%
	Yes	1,054	6.3%
	Refused	25	0.1%
Approximate total family/household income (in south African rands)	Less than 1,000 per month	2,564	13.9%
	Between 1,001–5,000 per month	11,540	65.3%
	More than 5,001 per month	2,495	13.0%
	Do not know	1,063	6.2%
	Refused	300	1.6%
The past 4 weeks how often was there no food to eat of any kind in house	Often	10,701	58.5%
	Sometimes	2,117	11.5%
	Rarely	609	3.6%
	Never	4,466	26.0%
	Refused	69	0.3%
Resilience (CYRM-12)		18,296	51.43 (10.42)
Depression (CES-D5)		18,277	1.18 (2.26)
Alcohol consumption (AUDIT-C)		18,277	0.76 (1.77)
Substance use		18,277	0.18 (0.97)
Are you involved in any sports clubs?	No	15,467	85.2%
	Yes	2,810	14.8%
How many of respondent’s friends drink alcohol at least once a week.	All of my friends	1854	11.4%
	Some of my friends	4,817	27.5%
	None of my friends	9,377	48.3%
	I do not know	2,229	12.8%
How many of respondent’s friends use dagga at least once a week.	All of my friends	784	4.7%
	Some of my friends	2,361	13.8%
	None of my friends	12,810	68.0%
	I do not know	2,322	13.5%
HIV knowledge	Poorer knowledge	8,372	45.3%
	Better knowledge	9,905	54.7%
Physical IPV	No	7,156	87.0%
	Yes	1,005	13.0%
Sexual IPV	No	7,628	93.0%
	Yes	533	7.0%
Any IPV	No	6,898	83.5%
	Yes	1,263	16.5%

The mean scores (mean = 51.43, SD: 10.42) for resilience highlighted high levels of resilience (range: 12–60). The mean score on the depression scale was fairly low (mean = 1.18, SD: 2.26) indicating a lower prevalence of depressive symptoms (range: 0–15). Nearly half (48.3%) of all the AGYW indicated none of their friends drink alcohol, whereas more than two thirds (68.0%) of AGYW indicated that none of their friends do drugs. Just over one-tenth (13.0%) of AGYW experienced physical IPV and 7.0% experienced sexual IPV. Just less than one-fifth (16.5%) experienced sexual and physical IPV (Table 1).

Table 2 highlights bivariate relationships between numerous psychosocial variables and HIV disaggregated by age. We highlight statistically significant differences within the age groups. The mean resilience score was higher for HIV positive than HIV negative AGYW among 15-19-year olds (*M* = 51.47 vs. 51.86, *p* < 0.05), although the inverse was found for 20-24-year olds as HIV negative AGYW had a higher resilience score than HIV positive AGYW (51.18 vs. 50.56, *p* < 0.05). For the depression scores across the age categories all the HIV negative individuals had lower scores than the HIV positive AGYW the largest difference being for older AGYW (1.39 vs. 2.16, *p* < 0.05). For alcohol use, mean scores on the AUDIT scale were higher among HIV positive than HIV negative AGYW aged 20–24 years old (M = 1.11 vs. 1.38, *p* < 0.05). Mean substance use was higher among HIV negative AGYW than HIV positive for 15–19 year olds (0.16 vs. 0.12, *p* < 0.05), the reverse was found among AGYW 20–24 years old as HIV positive individuals had a higher mean score on the substance use variable than HIV negative AGYW (0.46 vs. 0.23, *p* < 0.05). For HIV knowledge prevention it was clear that HIV positive AGYW across all the age groups had better knowledge about how to prevent HIV than their HIV negative counterparts. The greatest difference was found among 12-14-year-old AGYW (53.5% vs. 40.5%, *p* < 0.05).

**Table 2:** Bivariate associations between psychosocial variables and HIV disaggregated by age.

Level		12–14 years old				15–19 years old				20–24 years old			
		HIV -		HIV +		HIV -		HIV +		HIV -		HIV +	
		*N*	*M* (SD)/%	*N*	*M* (SD)/%	*N*	*M* (SD)/%	*N*	*M* (SD)/%	*N*	*M* (SD)/%	*N*	*M* (SD)/%
Individual													
Mean Resilience (CYRM-12) scale		4,125	52.01_a_ (10.03)	45	52.46_a_ (7.20)	6,815	51.47_a_ (10.32)	183	51.86_b_ (9.59)	6,663	51.18_a_ (10.68)	465	50.56_b_ (10.64)
Mean Depression score (CES-D5) scale		4,123	0.65_a_ (1.60)	45	1.08_b_ (1.79)	6,808	1.13_a_ (2.17)	183	1.68_b_ (2.69)	6,653	1.39_a_ (2.42)	465	2.16_b_ (3.45)
Mean Alcohol use (AUDIT-C) scale		4,123	0.09_a_ (0.53)	45	0.06_a_ (0.32)	6,808	.65_a_ (1.60)	183	0.65_a_ (1.59)	6,653	1.11_a_ (2.12)	465	1.38_b_ (2.31)
Mean Substance use		4,123	0.03_a_ (0.38)	45	0.04_a_ (0.36)	6,808	0.16_a_ (0.87)	183	0.12_b_ (0.59)	6,653	0.23_a_ (1.12)	465	0.46_b_ (1.96)
Mean Relationship control scale		484	13.94_a_ (4.12)	3	13.14_a_ (4.71)	4,035	12.85_a_ (4.01)	114	12.42 _b_ (4.54)	5,676	12.32_a_ (4.12)	437	11.62_b_ (4.28)
HIV knowledge	Poorer knowledge	2433_a_	59.5	19_b_	46.5	3088_a_	45.7	68_b_	38.5	2625_a_	39.7	139_b_	31.4
	Better knowledge	1690_a_	40.5	26_b_	53.5	3720_a_	54.3	115_b_	61.5	4028_a_	60.3	326_b_	68.6
Physical IPV	No	355_a_	94.1	2^3^	100.0	2752_a_	89.1	75_b_	80.7	3693_a_	86.0	279_b_	77.5
	Yes	23_a_	5.9	0^3^	0.0	320_a_	10.9	16_b_	19.3	574_a_	14.0	72_b_	22.5
Sexual IPV	No	360_a_	95.0	2^3^	100.0	2879_a_	93.3	86_a_	94.2	3990_a_	93.1	311_b_	87.9
	Yes	18_a_	5.0	0^3^	0.0	193_a_	6.7	5_a_	5.8	277_a_	6.9	40_b_	12.1
Any IPV	No	347_a_	91.7	2^3^	100.0	2646_a_	85.4	73_b_	78.5	3565_a_	82.6	265_b_	74.0
	Yes	31_a_	8.3	0^3^	0.0	426_a_	14.6	18_b_	21.5	702_a_	17.4	86_b_	26.0
Family/home													
What is the approximate total family/household income including grants and after	< R1000 per month	725_a_	18.8	4_b_	6.5	977_a_	14.6	24_b_	12.9	772_a_	11.4	62_b_	13.3
	R1001-R5000 per month	2256_a_	56.6	28_b_	68.2	4138_a_	62.9	122_b_	69.8	4658_a_	70.5	338_b_	72.2
	> = R5001 per month	680_a_	16.1	9_a_	18.4	940_a_	13.6	21_b_	10.3	809_a_	11.6	36_b_	7.8
	Do not know	220_a_	5.8	2_b_	3.9	450_a_	7.2	11_b_	5.8	353_a_	5.5	27_b_	6.3
	Refused	110_a_	2.7	1_a_	3.0	114_a_	1.7	2_b_	1.3	71_a_	1.0	2_b_	0.3
In the past 4 weeks, how often did you or any member of your household go to sleep hungry	Often	0^2,3^	0.0	0^2,3^	0.0	153_a_	4.5	2_b_	1.1	272_a_	4.2	29_b_	6.7
	Sometimes	0^2,3^	0.0	0^2,3^	0.0	373_a_	11.0	18_b_	18.7	740_a_	11.0	50_a_	11.2
	Rarely	0^2,3^	0.0	0^2,3^	0.0	266_a_	8.4	5_b_	4.7	567_a_	8.8	49_b_	10.7
	Never	0^2,3^	0.0	0^2,3^	0.0	2613_a_	76.2	84_a_	75.6	5084_a_	76.0	337_b_	71.5
Been away from home for more than 1 month consecutively in the previous year	No	4012_a_	97.1	45	100.0	6467_a_	94.8	175_a_	94.8	6114_a_	91.4	404_b_	85.7
	Yes	109_a_	2.8	0	0.0	337_a_	5.1	8_a_	5.2	539_a_	8.5	61_b_	14.3
	Refused	4_a_	0.1	0	0.0	11_a_	0.2	0	0.0	10_a_	0.1	0	0.0
Peer													
How many of your friends drink alcohol at least once a week?	All of my friends	159_a_	4.0	1_b_	2.5	671_a_	10.7	18_b_	9.4	930_a_	15.3	75_a_	15.4
	Some of my friends	445_a_	11.5	4_b_	9.1	1902_a_	28.2	52_b_	29.9	2247_a_	33.5	167_b_	35.1
	None of my friends	2951_a_	69.9	34_a_	71.5	3448_a_	49.1	85_b_	44.9	2686_a_	38.6	173_a_	37.8
	I do not know	568_a_	14.6	6_a_	16.9	787_a_	12.0	28_b_	15.8	790_a_	12.6	50_b_	11.7
How many of your friends use marijuana at least once a week?	All of my friends	101_a_	2.4	0^3^	0.0	294_a_	4.7	7_b_	3.3	354_a_	5.8	28_a_	5.9
	Some of my friends	174_a_	4.3	2_b_	5.9	987_a_	14.9	32_b_	18.6	1094_a_	16.9	72_b_	15.3
	None of my friends	3313_a_	79.4	38_a_	79.4	4705_a_	67.9	119_b_	63.4	4329_a_	63.3	306_b_	65.2
	I do not know	535_a_	13.9	5_a_	14.7	822_a_	12.5	25_b_	14.6	876_a_	14.1	59_a_	13.6
School													
Are you currently in school	No	109_a_	2.4	0	0.0	871_a_	15.7	30_b_	23.6	0	0.0	0	0.0
	Yes	4016_a_	97.6	45	100.0	4498_a_	84.3	104_b_	76.4	0	0.0	0	0.0
Community													
Is there a safe place in your community for you and your friends to meet?	No	1951_a_	47.2	17_b_	37.5	3147_a_	46.4	80_b_	44.4	2988_a_	45.2	199_b_	42.8
	Yes	2174_a_	52.8	28_b_	62.5	3668_a_	53.6	103_b_	55.6	3675_a_	54.8	266_b_	57.2
Are you involved in any sports clubs?	No	3011_a_	72.6	27_b_	63.0	5717_a_	83.7	158_b_	85.5	6115_a_	91.7	439_b_	94.2
	Yes	1112_a_	27.4	18_b_	37.0	1091_a_	16.3	25_b_	14.5	538_a_	8.3	26_b_	5.8

Values in the same row and sub-table that do not share the same subscript are significantly different at *p* < 0.05 level in the two-sided test of equality for column proportions. The tests assume equal variances. Tests are adjusted for all pairwise comparisons within a row of each innermost sub-table using the Bonferroni correction. Cells with no subscript are not included in the test.

HIV negative AGYW experienced more relationship control in their relationships than HIV positive individuals this was most pronounced among 20-24-year olds (12.32 vs. 11.2, *p* < 0.05). With regards to physical IPV, HIV positive individual were more likely to experience physical IPV in the previous 12 months among 15-19-year olds (10.9% vs. 19.3%, *p* < 0.05) and among 20–24- year olds (14.0% vs. 22.5%, *p* < 0.05). For sexual IPV, HIV negative respondents were less likely to experience sexual IPV than HIV positive respondents among 20-24-year old age group (6.9 vs. 12.1, *p* < 0.05). Very similar results were found for overall IPV results with among 20-24-year olds with a significant difference between HIV negative and HIV positive (17.4% vs. 26.0%, *p* < 0.05).

With regards to family or household level variables the data highlight that among 20-24-year olds HIV negative were more likely to never experience food insecurity in the previous month than those who were HIV positive (76.0% vs. 71.5%, *p* < 0.05). While within the same age group HIV positive were more likely to experience food insecurity often than HIV negative respondents (6.7% vs. 4.2%, *p* < 0.05). Among 20-24-year-old respondents HIV positive respondents were more likely to have been away from home for more than 1 month in the previous 12 months than HIV negative individuals (14.3% vs. 8.5%, *p* < 0.05).

Results from the peer influence variables indicate that among 12-14-year-old girls, those who are HIV negative reported a higher proportion of friends who drank alcohol compared to HIV positive girls (4.0% vs. 2.5%, *p* < 0.05). A similar pattern was observed among older adolescent girls (10.7% vs. 9.4%, *p* < 0.05).

Additionally, HIV negative adolescent girls aged 15–19 were more likely to be currently attending school compared to their HIV positive peers in the same age group (84.3% vs. 76.4%, *p* < 0.05).

HIV positive AGYW across the age groups were more likely to indicate there is a safe place they can go to with their friends than HIV negative girls (62.5% vs. 52.8%, *p* < 0.05). HIV positive adolescent girls were more likely to engage in sports club activities than HIV negative girls aged 12–14 years old (37.0% vs. 27.4%, *p* < 0.05). The opposite was true for young women aged 20–24 years old as HIV negative women were more likely to be involved in sports clubs than HIV positive women (8.3% vs. 5.8%, *p* < 0.05).

Table 3 presents the results of the multiple hierarchical logistic regression. The overall model was not statistically significant for the young 12-14-year olds (Nagelkerke R2 = 0.029, *p* = 0.284). There were also no statistically significant systemic effects.

**Table 3:** Multiple hierarchical regression between psychosocial variables and HIV disaggregated by age.

Level	Determinant	Dependent variable: HIV status (0 = Negative, 1 = Positive)		
		12–14 years old	15–19 years old	20–24 years old
		AOR (95% CI)	AOR (95% CI)	AOR (95% CI)
Individual (Block 1)	Resilience (CYRM-12)	1.00 (0.97–1.04)	1.01 (0.99–1.03)	1.00 (0.99–1.01)
	Depression (CES-D5)	1.11 (0.95–1.29)	1.12*** (1.05–1.18)	1.08***(1.04–1.11)
	Alcohol use (AUDIT-C)	0.86 (0.40–1.85)	0.96 (0.86–1.08)	1.01 (0.96–1.06)
	Substance use	1.38 (0.66–2.31)	0.96 (0.78–1.17)	1.10**(1.03–1.17)
	HIV knowledge			
	Poor knowledge	ref	ref	ref
	Good knowledge	2.04*(1.10–3.80)	1.39*(1.01–1.91)	1.51***(1.22–1.88)
	IPV			
	No IPV experienced	ref	Ref	ref
	Any IPV	0.00 (0.00–0.00)	1.48 (0.85–2.56)	1.41*(1.08–1.85)
	Missing data	2.18 (0.51–9.25)	1.04 (0.75–1.45)	0.68**(0.54–0.86)
	Model *χ*^2^ (df)	9.71 (7)	21.452 (7)	83.391 (7)
	Nagelkerke *R*^2^	0.019	0.014	0.030
Family/home (Block 2)	Away from home			
	No	.	ref	ref
	Yes	.	0.79 (0.38–1.63)	1.52**(1.14–2.03)
	Food security	.		
	Often	.	ref	ref
	Sometimes	.	3.33 (0.76–14.64)	0.54*(0.33–0.89)
	Rarely	.	1.32 (0.25–6.96)	0.65 (0.39–1.07)
	Never	.	2.28 (0.55–9.44)	0.50** (0.33–0.77)
	Missing	.	1.82 (0.43–7.70)	.
	Model *χ*^2^ (df)	.	32.032 (12)	100.664 (11)
	Block *χ*^2^ (df)	.	10.58 (5)	17.273 (4)
	Nagelkerke *R*^2^ (*R*^2^ change)	.	0.021 (0.007)	0.037 (0.007)
Peer (Block 3)	Friends drink			
	All of my friends	ref	ref	ref
	Some of my friends	0.98 (0.11–9.38)	0.94 (0.51–1.74)	1.00 (0.72–1.38)
	None of my friends	1.30 (0.17–9.75)	1.00 (0.53–1.90)	0.95 (0.67–1.36)
	I do not know	1.32 (0.16–11.25)	1.62 (0.73–3.58)	0.94 (0.58–1.53)
	Friends take drugs			
	All of my friends	.	ref	ref
	Some of my friends	.	1.22 (0.48–3.06)	0.82 (0.49–1.37)
	None of my friends	.	0.96 (0.39–2.38)	1.09 (0.66–1.79)
	I do not know	.	0.88 (0.31–2.50)	1.11 (0.61–2.01)
	Model *χ*^2^ (df)	9.24 (9)	36.718 (18)	104.523 (17)
	Block *χ*^2^ (df)	0.28 (3)	4.687 (6)	3.859 (6)
	Nagelkerke *R*^2^ (*R*^2^ change)	0.020 (0.001)	0.024 (0.003)	0.038 (0.001)
School (block 4)	Currently attending school	.		
	No	.	ref	ref
	Yes	.	0.75 (0.47–1.18)	.
	Missing data	.	0.91 (0.57–1.46)	.
	Model *χ*^2^ (df)	.	38.648 (20)	.
	Block *χ*^2^ (df)	.	1.749 (2)	.
	Nagelkerke *R*^2^ (*R*^2^ change)	.	0.026 (0.002)	.
Community (block 5)	Safe place in community for you and friends to meet			
	No	ref	ref	ref
	Yes	1.30 (0.70–2.41)	1.12 (0.82–1.15)	1.03 (0.85–1.08)
	Attend a sports club			
	No	ref	ref	ref
	Yes	1.76 (0.96–3.25)	0.87 (0.56–1.35)	0.68 (0.45–1.03)
	Model *χ*^2^ (df)	14.26 (12)	39.338 (22)	108.254 (19)
	Block *χ*^2^ (df)	4.174 (2)	0.870 (2)	3.732 (2)
	Nagelkerke *R*^2^ (*R*^2^ change)	0.029 (0.009)	0.026 (0.000)	0.039 (0.001)
	*N*	4,168	6,980	7,108

AOR, Adjusted Odds Ratio; CI, 95% Confidence Interval; HIV, Human Immunodeficiency Virus; ref, reference category; *p* < 0.05; **p < 0.01; ***p < 0.001.

For the 15-19-year olds the overall model was statistically significant (Nagelkerke *R*^2^ = 0.026, *p* = 0.013). There was systemic level effects for the individual level (*p* = 0.003) only. Displaying depressive symptoms had increased odds of being HIV positive among the 15–19 year old age group of AGYW (AOR: 1.12, 95% CI: 1.05–1.18, *p* < 0.001).

For the 20-24-year olds the overall model was statistically significant (Nagelkerke *R*^2^ = 0.039, *p* < 0.001). There was systemic level effects for the family/home level (*p* < 0.001) and individual level (*p* < 0.001). Displaying depressive symptoms increased the odds of being HIV positive (AOR: 1.08, 95% CI: 1.04–1.11, *p* < 0.001). Increase substance use was associated with increased likelihood of being HIV positive (AOR: 1.10, 95% CI: 1.03–1.17, *p* < 0.01). Those who had good knowledge on how to prevent HIV were more likely to be HIV positive (AOR: 1.51, 9% CI: 1.22–1.88, *p* < 0.001). Those who experienced IPV were more likely to be HIV positive (AOR: 1.41, 95% CI: 1.08–1.85, *p* < 0.05). Being away from home for more than 1 month consecutively in the previous year was associated with a higher likelihood of being HIV positive (AOR: 1.52, 95% CI: 1.14–2.03, *p* < 0.01). Those individuals who ‘never’ experienced food insecurity (in other words were food secure) were less likely to be HIV positive than those who ‘often’ experienced food insecurity (AOR: 0.50, 95% CI: 0.33–0.77, *p* < 0.01).

## 4 Discussion

This study sought to characterize psychosocial factors associated with HIV risk among AGYW in South Africa. The key findings include the association of depressive symptoms with HIV status, especially among those aged 15–24 years, and the link between IPV and HIV risk in the 20–24 age group. Additionally, food insecurity and substance abuse were found to be significantly associated with HIV prevalence. Finally, AGYW living with HIV showed better knowledge of HIV prevention compared to their HIV-negative counterparts, which may reflect the success of HIV prevention interventions over the years. Substantial gains in reducing new HIV infections in South Africa are at risk due to a reduction in the resources directed toward the HIV response. The highest HIV incidence is found AGYW, therefore preventing HIV infection among AGYW remains a public health imperative. Furthermore, an improved understanding of the drivers of HIV in this group is critical for developing appropriate interventions.

Our findings indicate a lower overall prevalence of depressive symptoms among the two youngest adolescent groups (12–15 and 15–19 years old). However, depressive symptoms were significantly associated with being HIV positive in the 15–19 and 20–24-year-old groups. This correlation has been previously reported among girls aged 13 to 21 years (35). Prior research indicates that depression can hinder condom negotiation, increasing HIV risk (36), a finding consistent with our results. Given the cross-sectional nature of our study, causality cannot be inferred. It is plausible that HIV-positive status may contribute to depressive symptoms, especially as adolescents come to understand the long-term implications of the diagnosis. This may be particularly true among older adolescents and young adults who have had more time to internalize and be affected by the diagnosis. This may partly explain the lower reported depressive symptoms among HIV-positive girls aged 12–14. These findings highlight the importance of age-sensitive mental health interventions that consider both developmental stage and HIV status. Future longitudinal studies are warranted to disentangle the directionality of the relationship between psychosocial distress and HIV, and to explore how these interactions evolve over time.

For young women aged 20–24 years old, IPV was associated with HIV status. Specifically, our results showed that HIV negative respondents were less likely to experience sexual IPV than HIV positive respondents. A previous study showed that married women who had faced IPV from their partners were almost twice more likely to have tested HIV positive compared to married women who did not face violence (37). This association likely reflects known mechanisms through which IPV heightens HIV risk, including limited negotiation power and exposure to forced sex (38).

Data from our study adds value to the existing literature on the negative impact of family or household factors on AGYW risky sexual behavior. Our results highlighted that food insecurity is associated with HIV status. Food insecurity has consistently been linked to increased HIV vulnerability through transactional sex and lower condom use (39, 40). Similar findings were reported by Tsai et al. (40), where severe food insecurity was associated with reduced odds of condom use among sexually active women. Interventions targeting food insecurity may have beneficial implications for HIV prevention among AGYW.

Substance abuse was higher among HIV-positive young women than HIV negative young women aged 20–24 years old. Although the direction of the relationship is not certain given the nature of the data. Extensive research has found that individuals who use alcohol are at higher risk for engaging in a number of risky sexual behaviors, including reduced condom use, increased number of sexual partners, and involvement in sex exchange for money (41, 42). Substance use during sex impairs risk perception and increases unsafe behaviors (43, 44). The use of alcohol and drugs increases the probability for sexual risk behavior through impairment of judgment, disinhibition, and reduced pain sensitivity resulting in an increased risk of unsafe sexual practices (44).

Young women living with HIV across all age groups demonstrated better knowledge about HIV prevention compared to their HIV-negative counterparts, with the largest difference observed among 12-14-year-old AGYW (53.5% vs. 40.5%, *p* < 0.05). This finding likely reflects the effectiveness of targeted HIV prevention and counseling programs delivered post-diagnosis, which provide individuals living with HIV with more comprehensive information on prevention strategies. It also suggests that knowledge acquisition may be a consequence rather than a cause of HIV status, highlighting the potential for reverse causality in this cross-sectional study. However, despite improved knowledge among those living with HIV, persistent challenges remain in delivering comprehensive sexual and reproductive health education to all adolescents, particularly in school and community settings (45), contributing to suboptimal prevention knowledge among HIV-negative AGYW. Additionally, better knowledge does not always translate into safer behaviors, as structural and psychosocial barriers such as intimate partner violence, poverty, or stigma may limit the ability to act on this knowledge effectively. These complexities underscore the need for holistic interventions that go beyond knowledge dissemination to address underlying vulnerabilities.

Beyond individual and household-level factors, broader structural determinants such as gaps in the education system, community norms, and economic conditions critically shape HIV risk. These structural influences interact with psychosocial and behavioral factors to affect young women’s vulnerability and access to prevention services. Addressing these wider determinants is essential for comprehensive HIV prevention efforts.

The DREAMS (Determined, Resilient, Empowered, AIDS-free, Mentored, and Safe) initiative, launched in 2015 by PEPFAR, is a multi-sectoral program targeting adolescent girls and young women in high HIV burden areas across sub-Saharan Africa, including South Africa. DREAMS aims to reduce HIV incidence through a comprehensive package of evidence-based interventions addressing biological, behavioral, and structural drivers of HIV risk. These interventions include HIV testing and counseling, condom promotion, education support, economic strengthening, gender-based violence prevention, and community mobilization. The program has been implemented in the study districts for several years, which may have contributed to moderating the influence of structural factors on HIV risk observed in our data. While our analysis did not find significant associations between certain structural factors and HIV prevalence, it is important to consider the potential impact of the DREAMS intervention. DREAMS, as a structural intervention aimed at addressing key social and economic determinants of HIV risk, may have reduced the effect of these structural factors in the districts where it was implemented. DREAMS may have mitigated structural drivers of HIV, explaining their weaker associations in this study. Therefore, the absence of a strong relationship between structural factors and HIV prevalence in this study may reflect the positive impact of DREAMS, which could have moderated the expected effects of these factors on HIV risk.

A few limitations of the study should be noted before interpreting these data. While we hypothesize that psychosocial factors may increase the risk of HIV infection, it is equally plausible that a positive HIV status or HIV diagnosis could lead to an increase in depressive symptoms and experiences of IPV. Given the cross-sectional nature of this study, it is not possible to assess the temporal order of events, and hence the direction of causality that led to the observed associations between psychosocial factors and HIV. The cross-sectional study design limits the ability to draw conclusions about causality, and we acknowledge this limitation in interpreting the findings. Additionally, the psychosocial factors were self-reported, which may introduce desirability or recall bias. Similarly, household income was self-reported and may underestimate actual earnings, particularly in cases involving informal or non-disclosed sources such as transactional sex. Importantly, the dataset did not include a specific variable identifying whether AGYW were engaged in transactional or commercial sex work. This represents a limitation, as sex work is known to be associated with increased exposure to both substance use and HIV infection. The sample cannot represent the entire country, given that the sampling focused on areas where DREAMS interventions were implemented. There may also be differences between girls in DREAMS areas and those in non-DREAMS areas, particularly in terms of access to and the impact of interventions. While our study exclusively includes girls from DREAMS areas, our findings are most relevant to populations living in high HIV incidence areas where similar interventions are being implemented. Therefore, while the results may not be directly generalizable to all settings, they are likely generalizable to other high-incidence areas where DREAMS or similar comprehensive intervention programs are in place. Findings from the multiple hierarchical regression indicate that individual-level factors appear to have the biggest influence on HIV prevalence, followed by family/household-level factors. This suggests that HIV interventions targeted at AGYW are likely to be beneficial if they address individual HIV risk factors such as mental health challenges and IPV. However, it is important to note that the cross-sectional nature of this study limits the ability to establish causal relationships. While these psychosocial factors may contribute to HIV risk, it is also plausible that experiencing HIV could exacerbate mental health issues and IPV, creating a bidirectional relationship. Future longitudinal studies are needed to better understand the directionality of these associations and to inform more targeted, effective interventions. Given the limitations of our dataset, we propose considering HIV status as a predictor of psychosocial factors, acknowledging the difficulty in establishing a clear causal direction with perinatally acquired HIV present in the sample.

## 5 Conclusion

Our findings highlight the significant role of psychosocial and behavioral factors in shaping HIV risk among adolescent girls and young women (AGYW) in South Africa. The association between depressive symptoms and HIV positivity, particularly among younger adolescents, underscores the need for integrating mental health support into HIV prevention programs. Similarly, the increased HIV risk among older AGYW due to substance use and intimate partner violence (IPV) points to the importance of comprehensive interventions that address both behavioral and structural vulnerabilities.

HIV prevention strategies should adopt a holistic approach that not only promotes safe sexual behaviors but also tackles underlying psychosocial challenges. Interventions targeting AGYW must incorporate mental health screening, IPV prevention, and substance use reduction initiatives to effectively mitigate HIV risk.

## Data Availability

The data analyzed in this study is subject to the following licenses/restrictions: The datasets generated during and/or analyzed during the current study are not publicly available, but are available from the corresponding author on reasonable request. Requests to access these datasets should be directed to becketts@ukzn.ac.za.

## References

[1] Risher KA Cori A Reniers G Marston M Calvert C Crampin A. Age patterns of HIV incidence in eastern and southern Africa: a modelling analysis of observational population-based cohort studies. Lancet HIV. (2021) 8:e429–39. doi: 10.1016/S2352-3018(21)00069-2, PMID: 34197773 PMC8258368

[2] UNAIDS. *AIDSinfo: Global data on HIV epidemiology and response*. Available online at: https://aidsinfo.unaids.org/ (Accessed November 15, 2022).

[3] Simbayi L Zuma K Zungu N. South African national HIV prevalence, incidence, behaviour and communication survey, 2017: towards achieving the UNAIDS 90–90-90 targets. Cape Town: HSRC (2019).

[4] Ramjee G Moonsamy S Abbai NS Wand H. Individual and population level impact of key HIV risk factors on HIV incidence rates in Durban, South Africa. PLoS One. (2016) 11:e0153969. doi: 10.1371/journal.pone.0153969, PMID: 27104835 PMC4841582

[5] Higgins JA Hoffman S Dworkin SL. Rethinking gender, heterosexual men, and women's vulnerability to HIV/AIDS. Am J Public Health. (2010) 100:435–45. doi: 10.2105/AJPH.2009.159723, PMID: 20075321 PMC2820057

[6] Magadi MA. The disproportionate high risk of HIV infection among the urban poor in sub-Saharan Africa. AIDS Behav. (2013) 17:1645–54. doi: 10.1007/s10461-012-0217-y, PMID: 22660933 PMC3663197

[7] Mabaso M Makola L Naidoo I Mlangeni LL Jooste S Simbayi L. HIV prevalence in South Africa through gender and racial lenses: results from the 2012 population-based national household survey. Int J Equity Health. (2019) 18:167. doi: 10.1186/s12939-019-1055-6, PMID: 31666077 PMC6821038

[8] Hallman K. Gendered socioeconomic conditions and HIV risk behaviours among young people in South Africa. Afr J AIDS Res. (2005) 4:37–50. doi: 10.2989/16085900509490340, PMID: 25865640

[9] Mufune P. Poverty and HIV/AIDS in Africa: specifying the connections. Soc Theor Health. (2015) 13:1–29. doi: 10.1057/sth.2014.14

[10] Niëns L Lowery D. Gendered epidemiology: sexual equality and the prevalence of HIV/AIDS in sub-Saharan Africa*. Soc Sci Q. (2009) 90:1134–44. doi: 10.1111/j.1540-6237.2009.00650.x

[11] Gottert A Barrington C McNaughton-Reyes HL Maman S MacPhail C Lippman SA. Gender norms, gender role conflict/stress and HIV risk behaviors among men in Mpumalanga, South Africa. AIDS Behav. (2018) 22:1858–69. doi: 10.1007/s10461-017-1706-9, PMID: 28161801 PMC6440537

[12] UNICEF. UNESCO Fixing the Broken Promise of Education for All: Findings from the Global Initiative on Out-of-School Children Montreal UNESCO Institute for Statistics. New York: UNICEF (2015).

[13] Sabates R Akyeampong K Westbrook J. School dropout: Patterns, causes, changes and policies. Education for All Global Monitoring Report. Paris: UNESCO (2011).

[14] Pettifor AE Levandowski BA Mac Phail C Padian NS Cohen MS Rees HV. Keep them in school: the importance of education as a protective factor against HIV infection among young south African women. Int J Epidemiol. (2008) 37:1266–73. doi: 10.1093/ije/dyn131, PMID: 18614609 PMC2734068

[15] Hardee K Gay J Croce-Galis M Afari-Dwamena NA. What HIV programs work for adolescent girls? J Acquir Immune Defic Syndr. (2014) 66:S176–85. doi: 10.1097/QAI.0000000000000182, PMID: 24918593

[16] UNAIDS. Empower young women and adolescent girls: Fast-tracking the end of the aids epidemic in Africa. Geneva: UNAIDS and the African Union (2015).

[17] Kuehner C. Why is depression more common among women than among men? Lancet Psychiatry. (2017) 4:146–58. doi: 10.1016/S2215-0366(16)30263-2, PMID: 27856392

[18] Barhafumwa B Miller CL Gray G Dietrich J Closson K Samji H. High prevalence of depression symptomology among adolescents in Soweto, South Africa associated with being female and cofactors relating to HIV transmission. Vulnerable Child Youth Stud. (2016) 11:263–73. doi: 10.1080/17450128.2016.1198854

[19] Nduna M Jewkes RK Dunkle KL Jama Shai NP Colman I. Prevalence and factors associated with depressive symptoms among young women and men in the eastern Cape Province, South Africa. J Child Adolesc Ment Health. (2013) 25:43–54. doi: 10.2989/17280583.2012.731410, PMID: 25860306

[20] Govender D Naidoo S Taylor M. Antenatal and postpartum depression: prevalence and associated risk factors among adolescents' in Kwa Zulu-Natal, South Africa. Depress Res Treat. (2020) 2020:2020: 5364521. doi: 10.1155/2020/5364521, PMID: 32411457 PMC7204344

[21] Larsen A Kinuthia J Lagat H Sila J Abuna F Kohler P. Depression and HIV risk behaviors among adolescent girls and young women seeking family planning services in Western Kenya. Int J STD AIDS. (2020) 31:652–64. doi: 10.1177/0956462420920423, PMID: 32538330 PMC7520985

[22] Nduna M Jewkes RK Dunkle KL Shai NP Colman I. Associations between depressive symptoms, sexual behaviour and relationship characteristics: a prospective cohort study of young women and men in the eastern cape, South Africa. J Int AIDS Soc. (2010) 13:44–4. doi: 10.1186/1758-2652-13-44, PMID: 21078150 PMC2992477

[23] Rane MS Hong T Govere S Thulare H Moosa MY Celum C. Depression and anxiety as risk factors for delayed care-seeking behavior in human immunodeficiency virus–infected individuals in South Africa. Clin Infect Dis. (2018) 67:1411–8. doi: 10.1093/cid/ciy309, PMID: 29659757 PMC6186861

[24] Berry MS Johnson MW. Does being drunk or high cause HIV sexual risk behavior? A systematic review of drug administration studies. Pharmacol Biochem Behav. (2018) 164:125–38. doi: 10.1016/j.pbb.2017.08.009, PMID: 28843425 PMC5747990

[25] Leigh BC Stall R. Substance use and risky sexual behavior for exposure to HIV. Issues in methodology, interpretation, and prevention. Am Psychol. (1993) 48:1035–45. doi: 10.1037/0003-066X.48.10.1035, PMID: 8256876 PMC2585544

[26] Malow RM Dévieux JG Jennings T Lucenko BA Kalichman SC. Substance-abusing adolescents at varying levels of HIV risk: psychosocial characteristics, drug use, and sexual behavior. J Subst Abus. (2001) 13:103–17. doi: 10.1016/s0899-3289(01)00069-4, PMID: 11547612

[27] Rehm J Shield KD Joharchi N Shuper PA. Alcohol consumption and the intention to engage in unprotected sex: systematic review and meta-analysis of experimental studies. Addiction. (2012) 107:51–9. doi: 10.1111/j.1360-0443.2011.03621.x, PMID: 22151318

[28] Mabaso M Sokhela Z Mohlabane N Chibi B Zuma K Simbayi L. Determinants of HIV infection among adolescent girls and young women aged 15-24 years in South Africa: a 2012 population-based national household survey. BMC Public Health. (2018) 18:183. doi: 10.1186/s12889-018-5051-3, PMID: 29373958 PMC5787232

[29] George G Cawood C Puren A Khanyile D Gerritsen A Govender K. Evaluating DREAMS HIV prevention interventions targeting adolescent girls and young women in high HIV prevalence districts in South Africa: protocol for a cross-sectional study. BMC Womens Health. (2020) 20:1–11. doi: 10.1186/s12905-019-0875-2, PMID: 31948429 PMC6966796

[30] Liebenberg L Ungar M Vijver FV. Validation of the child and youth resilience measure-28 (CYRM-28) among Canadian youth. Res Social Work Pract. (2012) 22:219–26. doi: 10.1177/1049731511428619

[31] Radloff LS. The CES-D scale: a self-report depression scale for research in the general population. Appl Psychol Meas. (1977) 1:385–401. doi: 10.1177/014662167700100306

[32] Bradley KA DeBenedetti AF Volk RJ Williams EC Frank D Kivlahan DR. Audit-c as a brief screen for alcohol misuse in primary care. Alcohol Clin Exp Res. (2007) 31:1208–17. doi: 10.1111/j.1530-0277.2007.00403.x, PMID: 17451397

[33] Jewkes RK Dunkle K Nduna M Shai N. Intimate partner violence, relationship power inequity, and incidence of HIV infection in young women in South Africa: a cohort study. Lancet. (2010) 376:41–8. doi: 10.1016/S0140-6736(10)60548-X, PMID: 20557928

[34] Statistics South Africa. *Mid year population estimates 2018*. Statistics South Africa (2018).

[35] Goin DE Pearson RM Craske MG Stein A Pettifor A Lippman SA. Depression and incident HIV in adolescent girls and young women in HIV prevention trials network 068: targets for prevention and mediating factors. Am J Epidemiol. (2019) 189:422–32. doi: 10.1093/aje/kwz238, PMID: 31667490 PMC7306677

[36] Klein H Elifson KW Sterk CE. Depression and HIV risk behavior practices among at risk women. Women Health. (2008) 48:167–88. doi: 10.1080/03630240802313605, PMID: 19042215 PMC6192253

[37] Shri N Muhammad T. Association of intimate partner violence and other risk factors with HIV infection among married women in India: evidence from National Family Health Survey 2015-16. BMC Public Health. (2021) 21:2105. doi: 10.1186/s12889-021-12100-0, PMID: 34789185 PMC8597306

[38] Maman S Campbell J Sweat MD Gielen AC. The intersections of HIV and violence: directions for future research and interventions. Soc Sci Med. (2000) 50:459–78. doi: 10.1016/S0277-9536(99)00270-1, PMID: 10641800

[39] Pascoe SJ Langhaug LF Mavhu W Pascoe SJS Hargreaves J Jaffar S. Poverty, food insufficiency and HIV infection and sexual behaviour among young rural Zimbabwean women. PLoS One. (2015) 10:e0115290. doi: 10.1371/journal.pone.0115290, PMID: 25625868 PMC4307980

[40] Tsai AC Bangsberg DR Frongillo EA Hunt PW Muzoora C Martin JN. Food insecurity, depression and the modifying role of social support among people living with HIV/AIDS in rural Uganda. Soc Sci Med. (2012) 74:2012–9. doi: 10.1016/j.socscimed.2012.02.033, PMID: 22513248 PMC3348339

[41] Chawla N Sarkar S. Defining “high-risk sexual behavior” in the context of substance use. J Psychosexual Health. (2019) 1:26–31. doi: 10.1177/2631831818822015

[42] Oppong Asante K Meyer-Weitz A Petersen I. Substance use and risky sexual behaviours among street connected children and youth in Accra, Ghana. Subst Abuse Treat Prev Policy. (2014) 9:45. doi: 10.1186/1747-597X-9-45, PMID: 25428774 PMC4258041

[43] Nadeau L Truchon M Biron C. High-risk sexual behaviors in a context of substance abuse: a focus group approach. J Subst Abus Treat. (2000) 19:319–28. doi: 10.1016/s0740-5472(00)00127-6, PMID: 11166496

[44] Ramsey SE Bell KM Engler PA. Human immunodeficiency virus risk behavior among female substance abusers. J Addict Dis. (2010) 29:192–9. doi: 10.1080/10550881003684756, PMID: 20407976 PMC2858863

[45] George G Tucker LA Panday S Khumalo F. Challenges facing life orientation educators in the delivery of sexuality education in south African schools. S Afr Rev Educ Educ Prod. (2018) 24:43–57. Available at: https://journals.co.za/doi/abs/10.10520/EJC-15ad838830

